# Anxiety, fatigue, and attentional bias toward threat in patients with hematopoietic tumors

**DOI:** 10.1371/journal.pone.0192056

**Published:** 2018-02-05

**Authors:** Kohei Koizumi, Jun Tayama, Toshiyuki Ishioka, Hiromi Nakamura-Thomas, Makoto Suzuki, Motohiko Hara, Shigeru Makita, Toyohiro Hamaguchi

**Affiliations:** 1 Department of Occupational Therapy, Graduate School of Health Science, Saitama Prefectural University, Koshigaya, Saitama, Japan; 2 Department of Cardiac Rehabilitation, Saitama International Medical Center, Saitama Medical University, Hidaka, Saitama, Japan; 3 Graduate School of Education, Nagasaki University, Nagasaki, Nagasaki, Japan; 4 School of Health and Social services, Saitama Prefectural University, Koshigaya, Saitama, Japan; Leiden University, NETHERLANDS

## Abstract

Cancer patients with hematopoietic tumors exhibit particularly high rates of anxiety disorders and depression, and often develop negative affect. In addition, psychological problems experienced by cancer patients impair their quality of life. When cancer patients feel anxious, they tend to direct their attention toward stimuli associated with threat in the surrounding environment. If attentional bias occurs in patients with hematopoietic tumors, who are at particular risk of developing negative affect, resolution of the bias could be useful in alleviating their anxiety. The current study examined the association between attentional bias and negative affect in patients with hematopoietic tumors and tested the hypothesis that negative affect would be more severe in those who exhibited greater attentional bias. Twenty-seven patients with hematopoietic tumors participated in the study. Reaction time (RT) was measured as the time between the presentation of the threatening and neutral images, and the subject’s button press to indicate choice (neutral expressions). Eight combinations of “threatening” expressions with high emotional valence and “neutral” expressions with low emotional valence were presented. The images used to measure attentional bias were taken from the Japanese Female Facial Expression Database and had been rated as expressive of anger, sadness, or neutrality, with predetermined emotional valence. Psychological testing was performed with the Profile of Mood States (POMS). To examine the association between attentional bias and negative affect, we calculated Spearman's rank correlation coefficients for RTs and POMS. Subjects’ mean RT was 882.9 (*SD* = 100.9) ms, and 19 of the 27 subjects exhibited slower RTs relative to healthy individuals. RT was significantly positively correlated with Tension-Anxiety (*r* = .679, *p* < .01) and Fatigue (*r* = .585, *p* < .01) subscale scores. The results of the study suggested that attentional bias toward threatening expressions could be positively correlated with the mental intensity of anxiety and fatigue in patients with hematopoietic tumors.

## Introduction

Cancer patients are known to be susceptible to reactive depression and anxiety [1], and depression and adjustment disorder are seen in 30–50% of this population [2–5]. In addition, cancer-related health worries are a significant predictor of both depression and anxiety in long-term survivors [6]. Cancer patients with hematopoietic tumors exhibit particularly high rates of anxiety disorder and depression, and often develop negative affect [7]. In addition, psychological problems experienced by cancer patients impair their quality of life [8].

Aerobic exercise during hospitalization was significantly correlated with quality of life (QOL), including physical and psychological well-being, depression, anxiety, and days hospitalized in patients with hematopoietic tumors [9]. In addition, music therapy and psychological counseling following a cognitive-behavioral approach including progressive muscle relaxation and cognitive techniques focusing on coping processes have been shown to reduce mood disturbance in these patients [10–11].

However, evidence and recommendations are still scarce for psychological interventions for patients with hematopoietic tumors, because of a lack of randomized controlled trials [12]. In addition, methods of psychological intervention to treat negative affect of patients with tumors has not shown progress with medical technological changes [1, 6]. Thus, the psychological adaptation of patients with cancer is a theme that should be investigated in oncological care.

When individuals feel anxious, they tend to direct their attention toward stimuli associated with threat in the surrounding environment [13]. This selective attention to threatening stimuli is more pronounced in highly anxious individuals [14] and known as “attentional bias” [15]. Attentional bias can be measured using a dot-probe task to evaluate spatial attention [16–18]. In one version of the dot-probe task, a threatening word or expression is presented and followed rapidly by a neutral stimulus positioned to one side, above, or below the original stimulus. A dot is then presented briefly in the position of one of the stimuli, and the subject is instructed to use a probe to indicate the location of the dot by pressing a button. The time taken to press the button once the dot has disappeared is measured as the subject’s RT, and longer RTs for neutral stimuli indicate that the subject's attention was selectively directed toward threatening stimuli [15, 19].

Attentional bias is a factor in the emergence and maintenance of anxiety and depression [20]. Attentional bias in chronic pain may not rely on preattentive processes, which, in the fear and anxiety literature, are hypothesized to play a key role in the fast detection of threats in the environment [21]. Attentional bias toward both pictorial and linguistic health-threat stimuli is present in individuals with chronic fatigue syndrome [22]. Moreover, selective attention to salient affective stimuli plays a role in depression and anxiety [21, 23]. A study using a Stroop task with cancer-related stimuli showed that women with the stress of having a family history of breast cancer exhibited greater interference on a task than women without cancer in the family [24]. On the other hand, survivors with clinical fear of cancer recurrence (FCR) had significantly greater positive beliefs about worry and beliefs about the uncontrollability and danger of worry than those with non-clinical FCR, while no significant differences were reported between participants’ FCR levels for attentional bias indices [25]. Accordingly, it is still unknown whether attentional bias acts as mechanism of negative emotion causality in cancer patients.

If attentional bias occurs in patients with hematopoietic tumors, who are at particular risk of developing negative affect, resolution of the bias could be useful in alleviating their anxiety. The current study (1) examined the association between attentional bias and negative affect in patients with hematopoietic tumors and (2) tested the hypothesis that negative affect would be more severe in those who exhibited greater attentional bias.

## Materials and methods

### Subjects

The study subjects included patients with hematopoietic tumors who had been admitted to Saitama Medical University International Medical Center and informed of their condition at least 2 weeks prior to the study. They had been diagnosed with leukemia or malignant lymphoma, were scheduled to undergo either chemotherapy or hematopoietic stem cell transplantation, had been prescribed occupational therapy, and were capable of sitting up for at least 40 minutes. Patients who had been diagnosed with a concomitant central nervous disorder, reported a history of depression or anxiety prior to their current illness, shown cognitive functional impairment (Mini Mental Scale Examination score of <24), or experienced severe autonomic nervous side effects during chemotherapy were excluded from the study [26].

Written informed consent was obtained from all the subjects prior to initiation of the study. This study was approved by the ethics committee at Saitama Prefectural University (approval no. 27506, October 19^th^, 2015) and the institutional review board at Saitama Medical University International Medical Center (approval no. 15–138, September 18^th^, 2015) and registered with the University Hospital Medical Information Network (UMIN000022141, April 29^th^, 2016) prior to commencement.

### Materials

Attentional bias was measured using attention bias modification (ABM) training software (ideoquest) installed on a laptop computer (Let's note LX3 CF-LX3, Panasonic). A 14-inch liquid crystal display (LCD) screen was used to display the stimuli. The subjects used the ABM training button-based input device to choose instantaneously between two images displayed on the screen. The distance between the subject's face and the LCD screen was approximately 45 cm.

### Measures

RT, which was used as an index of attentional bias, was measured as the time between the presentation of the two images via the ABM training software and the subject’s button press to indicate choice, using the computer's internal clock. Stimuli for the task consisted of pairs of facial expressions that contained one affective (angry or sad) and one neutral photograph. Participants were instructed to indicate as quickly as possible the location of the neutral face (above vs. below the screen) using the button. The computer recorded the RTs for each response. RTs of <200 ms or >2,000 ms were excluded from the statistical analysis as outliers [27].

To assess the association between negative affect and attentional bias, we performed psychological testing using the Japanese version of the Profile of Mood States (POMS) [28]. The reliability of the Japanese version of the POMS was examined in a healthy population, its validity was examined in people with depression, and an extended sample was used for standardization [28–30]. The POMS consists of six mood-state factors constituting negative affect, represented by six subscales: Tension-Anxiety, Depression-Dejection, Anger-Hostility, Activity, Fatigue, and Confusion. The raw scores for these subscales were used as psychological indicators. In the POMS, subjects are asked to complete 65 items pertaining to how they felt during the preceding week, with responses provided using a five-point scale ranging from 0 (*not at all*) to 4 (*extremely*). POMS scores were compared with the mean values and standard deviations for healthy individuals: scores equal to the mean ± 1 *SD* were considered healthy; scores equal to the mean ± 1–2.5 *SD*s indicated a possible requirement for examination by a medical specialist, to be decided in conjunction with other complaints; and scores equal to the mean ± >2.5 *SD*s indicated a definite requirement for examination by a medical specialist. In addition, we used Performance Status as an indicator of the general condition of cancer patients. These scales and criteria are used by doctors and researchers to assess how a patient’s disease is progressing, assess how the disease affects the daily living abilities of the patient, and determine appropriate treatment and prognosis [31].

### Procedure

The measurement of attentional bias and psychological assessment lasted approximately 20 min and was performed in the wards or at the rehabilitation center at Saitama Medical University International Medical Center.

The subjects completed the Japanese version of the POMS. Thereafter, they were asked to sit at a distance of 45 cm from an LCD screen, which was used for stimulus presentation. The task was explained to the subjects, and they were instructed to indicate the location of the target stimulus as accurately as possible, using the index finger or thumb of the dominant hand to press the input button. Attentional bias measurement took approximately 7 min.

### Task and stimuli

The images used to measure attentional bias were taken from the Japanese Female Facial Expression Database and had been rated as expressive of anger, sadness, or neutrality, with predetermined emotional valence [18, 32]. The correlation between these expressions and emotional valence has been demonstrated in a previous study [33]. Eight combinations of “threatening” expressions with high emotional valence and “neutral” expressions with low emotional valence were presented. The images were presented simultaneously, one above the other, at 1,600 (vertical) × 900 pixels (horizontal).

Subjects’ attentional bias was measured using a dot-probe task in which pairs of facial expressions, one threatening (i.e., angry or sad) and one neutral, with predetermined emotional valence, were displayed via the ABM training software. Subjects were required to press one of two input buttons to choose whether the neutral expression had been presented as the top or bottom image in the pair. The image stimuli consisted of eight types of display image presented 128 times in random order, and all of the subjects’ RTs were measured.

**Fig 1:**
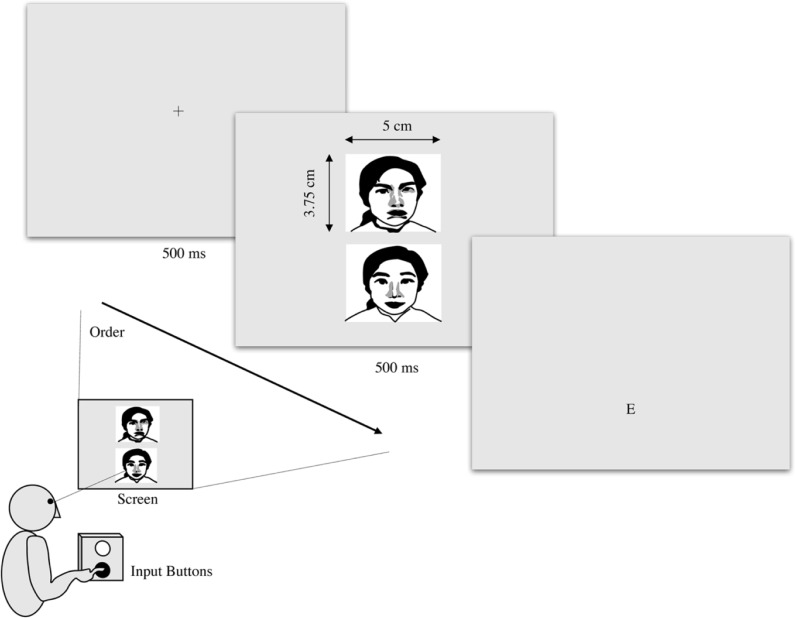
Procedure for measuring attentional bias. Once the facial photographs had been displayed for 500 ms, the subject was instructed to press a button to choose the most neutral face. The images were presented simultaneously, one above the other, at 1,600 (vertical) × 900 pixels (horizontal). The buttons could only be pressed after the images had been displayed, and the time taken to select the neutral image was recorded. Trials were performed 128 times, and RTs of <200 or >2,000 ms were excluded from the statistical analysis as outliers.

A fixation point was initially presented in the center of the LCD screen for 500 ms, followed by presentation of the two target images for 500 ms. The letter E was then presented for 500 ms, as a probe in place of the original neutral expression. Subjects were instructed to indicate the position of the neutral expression by pressing the selection button after the images had been displayed (Fig 1).

### Statistical analysis

To examine the association between attentional bias and negative affect, we calculated Spearman's rank correlation coefficients for the RTs recorded via the ABM training software and scores for the Tension-Anxiety, Depression-Dejection, Anger-Hostility, Activity, Fatigue, and Confusion subscales of the Japanese version of the POMS.

The subjects were divided into two groups according to the threshold of reported normal RTs, whether they exhibited high or low levels of attentional bias [34]. In the design to measure attentional biases, probes appear at the location of neutral stimuli. Thus, response latencies measure participants attention, with faster responses to targets in the attended location relative to the unattended location. Attentional bias towards threat is revealed when participants are slower to respond to probes that replace neutral-related stimuli, caused by attention to threat stimuli. The opposite pattern indicates avoidance of threat. In anxious individuals, schemas are thought to be biased toward threat [35]. The negative mood states in the Japanese version of the POMS that were significantly associated with attentional bias were compared between the two groups. An independent samples *t* test was performed to compare their scores for the six subscales of the Japanese version of the POMS. RTs of <200 ms or >2,000 ms were excluded from the statistical analysis as outliers, accounting for 1.56% of the total data. SPSS Version 24 (IBM) and JMP 13 were used to perform the statistical analysis, and the significance level was set at *p* < .05.

## Results

### Subjects’ characteristics

In total, 91 patients with hematopoietic tumors were admitted to Saitama Medical University International Medical Center between October 2015 and November 2016. Of these, 27 provided consent to participate in the study, and all 27 completed the study (Table 1). The subjects included seven women (mean age = 56, *SD* = 17 years) and 20 men (mean age = 61, *SD* = 17 years). Leukemia, myelodysplastic syndrome, non-Hodgkin’s lymphoma, aplastic anemia, and multiple myeloma were diagnosed in 10, four, eight, three, and two subjects (Table 2). None of the 27 patients who agreed to participate in the study expressed a desire to withdraw their participation. The approximate period from diagnosis to intervention was 2 months (mean days = 88, *SD* = 61).

**Table 1:** Demographic characteristics of participants.

Survey Items	Classifications	*n*	%
Sex	Male	20	74
	Female	7	26
Age	20–39	4	15
	40–49	3	11
	50–59	5	19
	60–69	6	22
	≥70	9	33
Marital status	Married/have a partner	22	81
	Single/divorced/separated/widowed	5	19
Highest education level	High school or below	14	52
	Vocational training/trade school	2	7
	University	11	41
Employment status	Paid employment	13	48
	Not in labour force	12	44
	Unemployed	2	7
Living arrangements	With others	26	96
	Alone	1	4
Smoking status	Former smoker	10	37
	Never smoked	17	63

**Table 2:** Disease and treatment characteristics of participants.

Survey Items	Classifications	*n*	%
Cancer Type	Non-Hodgkin lymphoma	8	30
	Leukemia	10	37
	Aplastic anemia	3	11
	Myelodysplastic syndromes	4	15
	Multiple myeloma	2	7
Cancer Stage	Early/less progression	15	56
	Late/more progression	12	44
Time since diagnosis	≤6 months	24	89
	7–12 months	3	11
	˃12 months	0	0
Recurrence	Yes	0	0
	No	27	100
Metastasis	Yes	0	0
	No	27	100
Treatment received	Chemotherapy only	22	81
	Chemotherapy and other treatment	5	19
	Other treatment only	0	0
Performance status*	0: Fully active	15	56
	1: Restricted in physically strenuous activity	7	26
	2: Up and about more than 50% of waking hours	5	18
	3: Capable of only limited self-care	0	0
	4: Completely disabled	0	0

*Performance Status is a measurement that describes a patient’s level of functioning in terms of their ability to care for themself, daily activity, and physical ability (walking, working, etc.).

### Mental state scores

Subjects' mean subscale scores for the Japanese version of the POMS were 17.0 (*SD* = 7.1) for Tension-Anxiety, 15.9 (*SD* = 9.5) for Depression-Dejection, 11.7 (*SD* = 6.4) for Anger-Hostility, 9.2 (*SD* = 4.5) for Activity, 10.6 (*SD* = 5.6) for Fatigue, and 10.0 (*SD* = 3.1) for Confusion (Table 3).

**Table 3:** Profile of Mood States (POMS) subscale scores in the study.

Subscale score	Mean (SD)	Number of abnormal score patients*
Tension-Anxiety	17.0 (7.1)	15
Depression	15.9 (9.5)	9
Anger-Hostility	11.7 (6.4)	6
Vigor	9.2 (4.5)	14
Fatigue	10.6 (5.6)	4
Confusion	10.0 (3.1)	4

*T-Score (T-score = 50 + 10 × (raw score − average) / standard deviation) was used for POMS abnormal score calculation. A T-score transformation produces a normal distribution with a mean of 50 and a standard deviation of 10. Abnormal scores are defined as T score ≤ 40 for "Vigor" and T score ≥ 60 for other subscales.

### Selective attention RTs

Subjects’ mean RT was 882.9 (*SD* = 100.9) ms.

### Association between attentional bias and mental state scores

**Fig 2:**
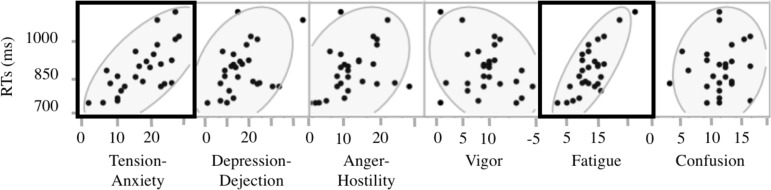
Analysis of the association between RTs and POMS scores. The horizontal axis shows POMS subscale scores and the vertical axis indicates RTs; scores for the 27 subjects are represented by dots in the gray regions. Bold boxes indicate significant correlations. There were significant positive correlations between RTs and scores for the Tension-Anxiety (*r* = 0.679, *p* < 0.01) and Fatigue (*r* = 0.585, *p* < 0.01) subscales. Correlation coefficients (*r*) were analyzed via Spearman's rank-order correlation analysis. *n* = 27, *p < .05.

RT was significantly positively correlated with Tension-Anxiety (*r* = .679, *p* < .01, 95% CI: 0.401–0.841) and Fatigue (*r* = .585, *p* < .01, 95% CI: 0.542–0.887) subscale scores (Fig 2).

### Differences in mental state scores between subjects with fast and slow RTs

**Fig 3:**
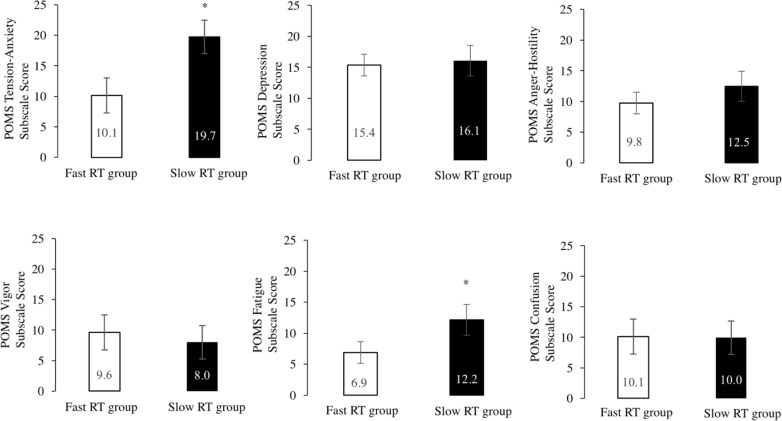
Comparison of negative POMS scores between subjects with fast and slow RTs. Mean RTs for the slower (*n* = 8) and faster (*n* = 19) groups were 778 (*SD* = 33) ms and 927 (*SD* = 86) ms, respectively. Both Tension-Anxiety (*t* = 4.108, *p* < .001, *r* = .64) and Fatigue (*t* = 2.724, *p* < .01, *r* = .48) subscale scores in subjects with slower RTs were significantly higher relative to those observed in subjects with faster RTs. Non-paired *t* test, *p < .05.

In accordance with a previous study [34], we divided the subjects into two groups according to whether their RTs were faster or slower relative to standard RT for healthy individuals (600–800 ms) and compared their scores for the subscales in the Japanese version of the POMS. The task of this study was similar to that previously used to assess attentional bias; threshold of RTs was 800 ms for dividing groups [34]. The results showed that eight subjects’ RTs were faster (*M* = 778, *SD* = 33 ms) and 19 subjects RTs were slower (*M* = 927, *SD* = 86 ms) relative to those for healthy individuals. A comparison of the mental states of these two groups indicated that scores for the Tension-Anxiety, *t* = 4.11, *p* < .001, *r* = 0.64, 95% CI: 1.41–12.50 and Fatigue, *t* = 2.72, *p* < .01, *r* = 0.48, 95% CI: 0.64–8.86 subscales in subjects with slow RTs were significantly higher relative to those observed for subjects with fast RTs (Fig 3). There was no significant difference between the two groups in scores for Performance Status (*p* < .594) and MMSE (*p* < .531).

## Discussion

In this study, patients with hematopoietic tumors were presented with images of threatening (angry or sad) and neutral expressions, and we measured their RTs in choosing the neutral expressions. We also analyzed the association between their mental states and attentional bias. Our results showed that RTs in making instantaneous decisions related to affect were longer in patients with hematopoietic tumors, and suggested that longer RTs were associated with increased anxiety and fatigue.

The standard RTs for healthy individuals were approximately 600–800 ms [27, 34]. The time between presentation of a visual stimulus and the firing of the neurons in the occipital lobe, which is responsible for visual information processing, is approximately 30 ms [36], while the time taken for reaction in the somatosensory area is approximately 150 ms [37]. The motor area is activated in anticipation of reaction to a stimulus, and once a stimulus has been processed, the passage of the signal from the motor area, via the corticospinal tract, to the finger muscles to initiate movement is believed to take approximately 20 ms [38]. Accordingly, the fastest time within which a human is able to process a visual stimulus and react via hand movement is approximately 200 ms. The selection of one of two images of facial expressions takes approximately 300 ms between stimulus presentation and initiation of information processing in the frontal lobe; thereafter, based on the results of this processing, a reaction or movement is chosen, and a motor command is transmitted to the muscles to generate a reaction [39].

According to a study involving functional magnetic resonance imaging of amygdala activity in response to neutral and threatening expressions, output from the amygdala increased in response to threatening stimuli [40]. In addition, greater activity has been observed in the fusiform gyrus (a region involved in facial cognition) in response to the presentation of images of sad facial expressions, relative to that observed for images of neutral expressions, and visual information processing associated with the amygdala has been found to begin approximately 170 ms subsequent to image presentation [41]. However, when the amygdala is active, threatening images are processed within approximately 200 ms [42], and the amygdala is known to be involved in emotional memory [43]. In addition, highly anxious individuals have been shown to exhibit elevated amygdala activity and a tendency to direct their attention toward threatening stimuli [17]. Moreover, Bishop, Duncan, Brett, and Lawrence (2004) reported that the prefrontal area was activated during the process of avoiding threatening expressions, and higher levels of state anxiety were associated with lower levels of activity in the prefrontal area [44]. The longer RTs observed in patients with hematopoietic tumors in the current study could have occurred because of patients’ anxiety, as amygdala activity was involved in the processing of emotional information required to recognize threatening expressions, which increased RTs.

In a psychological screening study of 10,153 cancer patients, analysis according to cancer type showed that patients with lung cancer, gynecological cancer, or hematopoietic tumors experienced significantly greater mental burden at the point of diagnosis [45]. In addition, another study examining the prevalence of anxiety and depression in 428 patients with hematopoietic tumors showed that 27% and 17% experienced anxiety disorder and depression, respectively [7]. Functional neuroimaging studies have been used to assess the relation between processing biases and functional brain response in patients with mood disorders. Results were altered neurophysiological responses in brain regions, such as the amygdala, hippocampus, and anterior cingulate cortex that process emotional information during tasks that utilize emotional stimuli [46]. Additionally, Mogg et al. reported that anxious individuals experienced neural excitement in the amygdala, which likely directed their attention to threat [17]. In the present study, the RTs observed in patients with hematopoietic tumors were positively correlated with scores for the Tension-Anxiety and Fatigue subscales of the Japanese version of the POMS. This finding suggested that higher levels of anxiety and fatigue were associated with greater attentional bias in patients with hematopoietic tumors.

In a study involving 17 healthy individuals, in which RTs were measured in a visual attention task after subjects had completed a task designed to induce mental fatigue, RTs increased significantly and were positively correlated with lower alpha power in electroencephalography [47]. An increase in alpha band power of the brain has been found to be related to a decrease in arousal, an increase in lower-alpha power is related to increased efforts (and probably difficulties) of subjects to maintain an attention. Moreover, in a comparative study of attentional bias in 14 chronic fatigue syndrome patients and 18 healthy individuals, a visual probe task using facial expression stimuli showed that patients with chronic fatigue syndrome exhibited longer RTs relative to those observed in healthy individuals [22, 48]. Hou et al. reported greater attentional bias for health-threat words than pictures and significantly impaired executive attention in 27 individuals with chronic fatigue syndrome [27]. A previous PET study demonstrated an underlying neuroinflamation in patients with chronic fatigue syndrome compared to healthy controls in widespread brain regions, especially the thalamus, midbrain, pons, amygdala, and anterior cingulate cortex [49]. In addition, in a study that evaluated mental fatigue in 281 cancer patients, using the Cancer Fatigue Scale, mental fatigue was strongly correlated with scores for the Japanese version of the POMS [50]. Therefore, fatigue in patients with hematopoietic tumors could have affected their RTs.

The basic strategy for improving attentional bias involves repeated reinforcement of neutral stimuli in an environment in which neutral stimuli and threatening stimuli that evoke negative affect are presented randomly, transforming attentional bias toward threatening stimuli and reducing anxiety. In a randomized controlled trial involving 40 pediatric patients with anxiety disorders, anxiety symptoms in patients who practiced ABM were significantly lower relative to those observed in individuals who did not practice the technique [51]. In addition, a systematic review of ABM showed that it exerted a significant effect on the reduction of anxiety [52]. Therefore, teaching patients with hematopoietic tumors to avoid directing their attention toward topics that evoke negative affect could aid in the alleviation of their anxiety.

There are some limitations to the current study. The subjects in the current study included patients with hematopoietic tumors, and their attentional bias was not compared to that of healthy individuals or other cancer patients as comparative controls. In addition, personality traits and behavioral characteristics are known to contribute to affect [53]; however, they were not examined in this study, to avoid inflicting excessive mental burden on subjects. However, if they contribute to attentional bias in patients with hematopoietic tumors, they should be considered in the development of ABM techniques that affect the psychological well-being of cancer patients during rehabilitation.

## Conclusions

The results of this study suggested that attentional bias toward threatening expressions could be positively correlated with the mental intensity of anxiety and fatigue in patients with hematopoietic tumors. Attentional bias could be involved in inducing negative affect in patients with hematopoietic tumors, and interventions to reduce this bias could aid in the alleviation of negative affect.
